# Profil clinique de l´incontinence urinaire de la femme aux Cliniques Universitaires de Kinshasa de 2015 à 2016: une étude descriptive rétrospective

**DOI:** 10.11604/pamj.2020.37.386.18036

**Published:** 2020-12-30

**Authors:** Andy-Müller Luzolo Nzinga, Inès Bilo Mbaki, Pompon Kazadi Ilunga, François Njimbu Kapend, Nadine Mbanzulu Diyasilua, Roger Mwimba Mbungu, Mathieu Nkumu Loposso, Betty Miangindula Mabenza, Augustin Mboko Kipula, Honoré Nkakudulu Bikuku

**Affiliations:** 1 Unité de Rééducation Uro-Gynécologique, Département de Médecine Physique et de Réadaptation, Cliniques Universitaires de Kinshasa, Faculté de Médecine, Université de Kinshasa, Kinshasa, République Démocratique du Congo,; 2 Ecole de Santé Publique, Faculté de Médecine, Université de Kinshasa, Kinshasa, République Démocratique du Congo,; 3 Service d´Obstétrique, Département de Gynécologie et Obstétrique, Cliniques Universitaires de Kinshasa, Faculté de Médecine, Université de Kinshasa, Kinshasa, République Démocratique du Congo,; 4 Service d´Urologie, Département de Chirurgie, Cliniques Universitaires de Kinshasa, Faculté de Médecine, Université de Kinshasa, Kinshasa, République Démocratique du Congo,; 5 Unité d´Activités Physiques Adaptées, Département de Médecine Physique et de Réadaptation, Cliniques Universitaires de Kinshasa, Faculté de Médecine, Université de Kinshasa, Kinshasa, République Démocratique du Congo

**Keywords:** Prise en charge, incontinence urinaire, femme, Cliniques Universitaires de Kinshasa, Management, urinary incontinence, woman, University Clinics of Kinshasa

## Abstract

L´incontinence urinaire (IU) de la femme est une pathologie très fréquente dans la population. La carence des données épidémio-cliniques dans notre milieu nous a poussés à faire un état de lieu de cette affection et de sa prise en charge aux Cliniques Universitaires de Kinshasa. L´objectif de cette étude est de déterminer la prévalence et la prise en charge de l´incontinence urinaire de la femme en milieu clinique. Une étude descriptive a été réalisée aux Cliniques Universitaires de Kinshasa de janvier 2015 à décembre 2016. La fréquence annuelle de l´IU était de 1,3% (soit 23/1813 patientes). Nous n´avons retenu que 15 cas dont les dossiers étaient exploitables, et leur âge moyen était de 49,2±20,5 ans avec des extrêmes de 15 à 98 ans. L´IU a concerné les multipares (53,4%), les paucipares (26,7%), les primipares (6,7%) et les nullipares (13,3%), et la date médiane de survenue de l´IU était de 3 mois. L´IU par urgenturie était présente chez 33,3% des patientes et celle à l´effort chez 13,3%. Parmi les diagnostics associés à l´IU, nous avons plus retrouvé les infections uro-génitales (46,7%), la cystocèle (20%) et les algies pelviennes chroniques (20%). Ces patientes ont bénéficié de l´antibiothérapie (60%), des anticholinergiques (20%), de la rééducation pelvipérinéale (20%) ainsi que du traitement chirurgical. L´IU est sous-évaluée aux Cliniques Universitaires de Kinshasa. Les types d´IU les plus diagnostiqués sont les IU à l´effort et par urgenturie. La prise en charge est pluridisciplinaire.

## Abstract (en)

Urinary Incontinence (UI) in women is a very common disease. Given the shortage of epidemiological and clinical data in our environment, we here provide an update on the status of this disease and its management at the University Clinics of Kinshasa. We conducted a descriptive study in the University Clinics of Kinshasa from January 2015 to December 2016. The annual rate of IU was 1.3% (23/1813 patients). We included 15 cases whose medical files were usable and whose average age was 49.2±20.5 years, ranging between 15 and 98 years. IU affected multiparous women (53.4%), pauciparous women (26.7%), primiparous women (6.7%) and nulliparous women (13.3%) and the median date of onset of IU was 3 months. Urge incontinence affected 33.3% of patients and effort incontinence 13.3%. Among diagnoses associated with UI, the most common were urogenital infections (46.7%), cystocele (20%) and chronic pelvic pain (20%). These patients received antibiotic therapy (60%), anticholinergics drugs (20%), and pelviperineal rehabilitation (20%) as well as surgical treatment. UI is underestimated at the University Clinics of Kinshasa. The most commonly diagnosed IUs are effort and urge incontinence. Patient´s management is based on multidisciplinary approach.

## Introduction

L´incontinence urinaire (IU) de la femme est une pathologie très fréquente. Elle est définie en 1979 par l´*International Continence Society (I.C.S)* comme étant la perte involontaire d'urine par l´urètre en dehors de toute miction, démontrée objectivement et constituant un problème social ou d'hygiène [1]. Plusieurs études traitent sur la prévalence de l´IU chez la femme, mais cette dernière varie en fonction du type d´étude, de la définition et de l´évaluation de l´IU ainsi que des caractéristiques de la population étudiée [2]. Lors de la 6^e^ édition de l´*International Consultation on Incontinence (ICI)* en 2017, il a été constaté, à travers 36 études menées dans 17 pays, que la prévalence de l´IU chez les femmes était de l'ordre de 5% à 69% [3]. L´étude « The prevalence of urinary incontinence in women in four European countries » a estimé les prévalences de l´IU chez des femmes de plus de 18 ans à 23% en Espagne, 41% en Allemagne, 42% en Grande Bretagne et 44% en France [4]. En Afrique, quelques études ont fait état de prévalence élevée, estimée à 22,3%; 41,5%; 21,3%; 31% et 10,8% respectivement au Maroc, au Ghana, au Burkina-Faso, au Sénégal et à la Mauritanie [5-8]. En République Démocratique du Congo (RDC), il n´existe pas, en notre connaissance, des données épidémio-cliniques publiées sur l´incontinence urinaire de la femme et sa prise en charge. Cette carence des données dans notre milieu nous a poussés à faire un état de lieu de cette affection et de sa prise en charge aux Cliniques Universitaires de Kinshasa. L´objectif de cette étude est de déterminer la prévalence et la prise en charge de l´incontinence urinaire de la femme en milieu clinique dans le but de contribuer à l´amélioration de cette prise en charge multidisciplinaire.

## Méthodes

Il s´agit d´une étude descriptive rétrospective réalisée sur 1813 dossiers des femmes qui ont consulté pendant la période de janvier 2015 à décembre 2016 aux Cliniques Universitaires de Kinshasa (CUK), dans les services de gynécologie générale et de maternité du Département de Gynéco-Obstétrique, dans le Service d´Urologie du Département de Chirurgie, et enfin à l´unité de rééducation d´uro-pelvipérinéale du Département de Médecine physique et réadaptation (Tableau 1). Sur un total de 1813 femmes qui ont consulté les CUK, il ressort de ce Tableau 1 que la fréquence moyenne annuelle de l´IU de la femme était de 1,3% au cours des années 2015 et 2016. Le Département de Gynécologie - Obstétrique avait moins diagnostiqué l´incontinence urinaire avec une fréquence de 0,6% (N=1688) tandis que le service d´urologie avait une fréquence de 4,3% (N=115). Sur les 10 femmes qui ont consulté l´unité de rééducation d´uro-pelvipérinéale, 8 soit 80% ont présenté une incontinence urinaire. Sur le total de 23 femmes qui ont été diagnostiquées d´incontinence urinaire, nous n´avons pu retenir que 15 cas dont les dossiers étaient exploitables pour notre étude. Et l´âge moyen de ces patientes était de 49,2±20,5 ans avec des extrêmes variant entre 15 à 98 ans. Le nombre de cas très faible dans cette étude s´expliquerait par le fait que c´est une affection non abordée soit par les médecins ou soit par les patientes elles-mêmes. Nous avons élaboré une fiche de collecte des données reprenant les variables telles que l´âge, la parité, types d´IU, la date de survenue de l´IU, les diagnostics associés à l´IU et les traitements administrés pour l´IU.

**Tableau 1:** fréquence de l´incontinence urinaire (IU) de la femme aux Cliniques Universitaires de Kinshasa

	Années						Total		
	2015			2016					
Lieux	Femmes consultées	Femmes avec IU	Fréquence de l'IU (%)	Femmes consultées	Femmes avec IU	Fréquence de l'IU (%)	Femmes consultées	Femmes avec IU	Fréquence de l'IU (%)
Services de Gynécologie-obstétrique	882	5	0,6	806	5	0,6	1688	10	0,6
Service d’urologie	82	2	2,4	33	3	9,1	115	5	4,3
Unité de rééducation d’uro-pelvipérinéale	5	5	100	5	3	60	10	8	80
**Total**	969	12	1,2	844	11	1,3	1813	23	1,3

## Résultats

**Parité:** l´IU était plus fréquente chez les multipares avec 53,4%, suivie des paucipares dans 26,7% des cas puis des nullipares dans 13,3% des cas. Et 6,7% des primipares ont aussi développé l´IU (Tableau 2).

**Tableau 2:** répartition des patientes selon la parité

Parité	Effectif	Pourcentage
Nullipare	2	13,3
Primipare	1	6,7
Paucipares (2-3)	4	26,7
Multipares (4-13)	8	53,4
**Total**	15	100

**Caractéristique de l´incontinence urinaire:** la date de survenue (ancienneté) de l´IU variait entre 2 semaines à 108 mois avec une médiane de 3 mois. Le Tableau 3 révèle que l´IU par urgenturie isolée était la plus représentée chez 33,3% des cas, suivie de l´IU mixte chez 20% des cas puis de l´IU à l´effort et de l´IU suite à une neurovessie dans 13,3% des cas. Parmi les diagnostics associés à l´IU, nous avons plus retrouvé les infections uro-génitales dans 46,7% des cas, la cystocèle dans 20% des cas et les algies pelviennes chroniques dans 20% des cas. Nous notons aussi la dysurie, la grossesse, le cancer du col utérin ainsi que le kyste périméatal de l'urètre dans 6,7% des cas chacun (Tableau 3).

**Tableau 3:** caractéristiques cliniques de l´IU

Clinique	Effectif		Pourcentage
**Types d'IU**			
IUM	3		20
IU par regorgement	1		6,7
IUU	5		33,3
Vessie neurogène	2		13,3
IU sur fistule vésico-vaginale	1		6,7
Enurésie nocturne	1		6,7
IUE	2		13,3
**Total**	15		100
**Diagnostics associés (N=15)**			
Dysurie	1		6,7
Infections uro-génitales	7		46,7
Cystocèle	3		20
Algies pelviennes chroniques	3		20
Grossesse	1		6,7
Cancer du col	1		6,7
Kyste perinatal de l'urètre	1		6,7

**Traitement administré:** les patientes incontinentes urinaires ont bénéficié d´antibiothérapie (60%), des anticholinergiques (20%) ainsi que de la rééducation pelvipérinéale (20%). Comme traitement chirurgical, nous avons noté l´urétérostomie bilatérale, la kystectomie périméatale urétrale et la pose de la sonde urinaire, dans une proportion de 6,7% chacune. Une patiente, soit 6,7%, n´avait bénéficié d´aucun traitement (Tableau 4).

**Tableau 4:** traitement administré chez les patientes incontinence urinaire

Traitement	Effectif	Pourcentage
Antibiotiques	9	60
Anticholinergiques	3	20
Sonde urinaire à demeure	1	6,7
urétérostomie bilatérale	1	6,7
kystectomie périméatale urétrale	1	6,7
Rééducation pelvipérinéale	3	20
Aucun traitement	1	6,7

## Discussion

L´IU fait référence à un symptôme, un signe clinique, une observation urodynamique ou un état pathologique [9]. Sa fréquence moyenne annuelle chez la femme était de 1,3% au cours des années 2015 et 2016. Cette dernière est faible, bien qu´elle rejoint presque les données de la littérature que ce soient pour des études cliniques ou soient pour des enquêtes dans la population dont elle varie entre 1,4% à 69% [2, 3]. Il faut noter qu´en milieu clinique, la prévalence de l´incontinence urinaire de la femme est moins bien connue. À partir de l´analyse des dossiers médicaux informatisés de 24 735 femmes de plus de 40 ans, les auteurs de ladite étude ont estimé le taux de prise en charge de l´incontinence urinaire à 1,4% des femmes ayant consulté au cours de l´année 1999 [10]. Et pour d´autres auteurs, la prévalence de l´IU chez les femmes en âge de procréation, entre 15 à 44 ans, était de 2,5% [11]. Les gynécologues et obstétriciens étaient les professionnels de santé qui avaient reçu plus des femmes en consultations dans notre série, mais néanmoins, l´IU était la moins diagnostiquée. Ceci prouve qu´ils n´abordent pas du tout ou pas systématiquement le sujet lors de la consultation, même devant les femmes à risque. Behague L. confirme dans son étude que l´IU n´est pas systématiquement recherchée par les médecins [12], surtout si la femme ne s´en plaint pas. Et parmi ces patientes diagnostiquées d´incontinence urinaire, chez presque eux toutes, la découverte était secondaire à d´autres motifs de consultation, parmi lesquels les infections uro-génitales, la cystocèle, et les algies pelviennes chroniques, la dysurie, la grossesse, le kyste périméatal de l'urètre ainsi que le cancer du col utérin entrainant une fistule vésico-vaginale. Ceci parce que les femmes n´en parlent pas spontanément et deviennent mal à l´aise lorsque vient le moment d´en parler à un médecin, car elle la considère comme un sujet tabou, honteux ou une fatalité lié à l´âge et à l´accouchement [12].

Cette affection a concerné toutes les femmes en âge de procréation, avec un âge moyen de 49,2 ans, et surtout les femmes qui ont déjà accouchées dépendant de la parité. D´après la littérature, l´IU augmente significativement et de façon linéaire avec l´âge, avec quelques variations en fonction du type de fuites [13]. Ainsi, le risque d´IU est retrouvé 3,6 fois plus élevé à partir de 30 ans [14]. Et pour l´Association Française d´Urologie, la fréquence et la sévérité de l´IU augmentent avec l´âge. Il existe deux pics, un premier à 45-50 ans au moment de la ménopause, un deuxième après 75 ans [15]. Aubin confirme dans son étude que la grossesse et l'accouchement par voie basse provoquent fréquemment une IU, par la distension importante des ligaments et du plancher pelvien qu'ils occasionnent [16]. Et dans une étude de cohorte, le nombre d´accouchements était un facteur de risque corrélé à la prévalence de l´IU à long terme [17]. Quant aux types d´IU, notre étude a révélé que l´IU par urgenturie était la plus représentée que l´IU à l´effort, ceux-ci contrairement à plusieurs études. Et nous avons aussi noté d´autres formes d´IU. Selon la majorité d´études menées, l´IU à l´effort est la plus fréquente, variant entre 10 - 54,2% tandis que l´IU par urgenturie est la moins représentée avec au taux variant entre 1 - 28,6% [6, 18]. Cette prédominance d´IU par impériosité dans notre étude peut s´expliquer par la proportion élevée d´infections urogénitales chez ces patientes qui ont consulté les Cliniques Universitaires de Kinshasa. La littérature incrimine l´infection urinaire à répétition dans la genèse de l´incontinence urinaire par impériosité [9, 19]. Les cliniciens distinguent, par conséquent, plusieurs types d´IU qui correspondent à une présentation clinique variable selon l´individu. Les types les plus fréquents sont l´IU d´effort et l´IU par urgenturie. Il est probable que chaque type d´IU soit la conséquence d´un ou de plusieurs mécanismes physiopathologiques spécifiques liés à l´exposition individuelle, à des facteurs de risque d´IU qui sont multiples, certains modifiables (le poids), d´autres non (le vieillissement), et qui peuvent avoir une influence variable selon le type d´incontinence [20]. Pour ce faire, l´incontinence urinaire ne doit plus être considérée comme un état inéluctable chez la femme, mais comme un état pathologique nécessitant consultation et prise en charge [15]. Elle peut être symptomatique d´autres pathologies, qui peuvent bénéficier d´un traitement spécifique [21].

Les dernières recommandations de l´Agence Nationale d´Accréditation et d´Evaluation en Santé (ANAES 2003) préconisent différents traitements en fonction du type d´IU. Ainsi, les traitements comportementaux (reprogrammation mictionnelle, tenue d´un calendrier mictionnel) sont recommandés dans le traitement de l´incontinence par urgenturie, la rééducation périnéo-sphinctérienne, seule ou associée au biofeedback ou à l´électrostimulation, est recommandée dans l´incontinence d´effort, les traitements anticholinergiques (l'oxybutynine, la toltérodine) dans l´incontinence urinaire par urgenturie, et des traitements chirurgicaux après échec d´une rééducation périnéo-sphinctérienne ou plus rarement d´emblée en cas d´IU à l´effort invalidante [2]. Dans notre étude, les traitements administrés chez ces patientes incontinentes étaient dirigés soit vers les facteurs étiologiques ou soit vers la symptomatologie. Ces approches étaient plus pharmacologiques que rééducationnelles, faites d´antibiothérapie et des anticholinergiques. Et nous avons noté quelques cas de chirurgie. Par manque d´issus thérapeutiques dans les dossiers médicaux, il est difficile d´apprécier l´efficacité de la prise en charge de l´incontinence urinaire de la femme aux Cliniques Universitaires de Kinshasa. L´antibiothérapie y était plus prescrite pour les cas d´infections urogénitales décelées comme la cause principale d´IU chez la femme. Et ceci est l´attitude ou le réflexe clinique de presque beaucoup des médecins devant cette affection dans nos milieux, bien que cela ne soit pas la cause principale de survenue de l´IU chez la femme. Ceci peut se confirmer par l´étude de Monz B *et al*. menée auprès des professionnels de santé où il se dégage qu´il se pose un problème de stratégie de dépistage de l´incontinence, car beaucoup sont désarmés devant les mécanismes physiopathologiques et la prise en charge de l´IU. Et aussi, ils se sont plaints d´un manque d´information sur les modalités de la rééducation en cas l´incontinence urinaire [22]. D´après l´ANAES de 2000, traitant sur les techniques de rééducation dans la prise en charge de l´IU, quelles que soient les pathologies et les techniques de rééducation utilisées, les études ont montré l´intérêt de la prise en charge rééducative en première intention [23].

## Conclusion

Bien que l´incontinence urinaire soit fréquente dans la population féminine, d´après plusieurs auteurs, elle est sous diagnostiquée aux Cliniques Universitaires de Kinshasa. Les femmes consultent plus pour autres problèmes de santé que pour l´IU, surtout plus pour infections urogénitales, étant donné qu´en parler reste un sujet tabou ou une fatalité pour elles. Cette affection touche toutes les femmes en âge de procréation et surtout les femmes qui ont déjà accouché. Il existe plusieurs types d´IU, mais les plus fréquentes sont les IU à l´effort et par urgenturie. Toutes les approchent thérapeutiques étaient indiquées mais l´approche médicamenteuse était la plus prescrite, surtout l´antibiothérapie. La rééducation a aussi fait partie de cette pluridisciplinarité bien qu´elle ne soit pas prescrite en première intention. Raison pour laquelle, les médecins doivent, si possible, aborder systématiquement ce sujet lors de consultation chez les femmes qui y viennent et de proposer une prise en charge pluridisciplinaire et efficace.

### Etat des connaissances sur le sujet

- L´incontinence urinaire (IU) de la femme est une pathologie très fréquente; elle est définie comme étant la perte involontaire d'urine par l´urètre en dehors de toute miction, démontrée objectivement et constituant un problème social ou d'hygiène;
- L´incontinence urinaire ne doit plus être considérée comme un état inéluctable chez la femme, mais comme un état pathologique nécessitant consultation et prise en charge;
- En RDC, il n´existe pas, en notre connaissance, des données épidémio-cliniques publiées sur l´incontinence urinaire de la femme et sa prise en charge.

### Contribution de notre étude à la connaissance

- Cette étude apporte des nouvelles informations sur la fréquence, la clinique ainsi que traitement administré aux femmes avec incontinence urinaire aux cliniques universitaires de Kinshasa en particulier, et aux milieux cliniques de la RDC.
